# Bromodomain Containing Protein 4 (BRD4) Regulates Expression of its Interacting Coactivators in the Innate Response to Respiratory Syncytial Virus

**DOI:** 10.3389/fmolb.2021.728661

**Published:** 2021-10-26

**Authors:** Xiaofang Xu, Morgan Mann, Dianhua Qiao, Yi Li, Jia Zhou, Allan R. Brasier

**Affiliations:** ^1^ Department of Medicine, University of Wisconsin-Madison School of Medicine and Public Health (SMPH), Madison, WI, United States; ^2^ Department of Pharmacology and Toxicology, University of Texas Medical Branch, Galveston, TX, United States; ^3^ Institute for Clinical and Translational Research (ICTR), University of Wisconsin-Madison, Madison, WI, United States

**Keywords:** BRD4, BRD4 (bromodomain-containing protein 4), interferon, ISG (interferon-stimulated genes), MMP-9

## Abstract

Bromodomain-containing protein 4 plays a central role in coordinating the complex epigenetic component of the innate immune response. Previous studies implicated BRD4 as a component of a chromatin-modifying complex that is dynamically recruited to a network of protective cytokines by binding activated transcription factors, polymerases, and histones to trigger their rapid expression via transcriptional elongation. Our previous study extended our understanding of the airway epithelial BRD4 interactome by identifying over 100 functionally important coactivators and transcription factors, whose association is induced by respiratory syncytial virus (RSV) infection. RSV is an etiological agent of recurrent respiratory tract infections associated with exacerbations of chronic obstructive pulmonary disease. Using a highly selective small-molecule BRD4 inhibitor (ZL0454) developed by us, we extend these findings to identify the gene regulatory network dependent on BRD4 bromodomain (BD) interactions. Human small airway epithelial cells were infected in the absence or presence of ZL0454, and gene expression profiling was performed. A highly reproducible dataset was obtained which indicated that BRD4 mediates both activation and repression of RSV-inducible gene regulatory networks controlling cytokine expression, interferon (IFN) production, and extracellular matrix remodeling. Index genes of functionally significant clusters were validated independently. We discover that BRD4 regulates the expression of its own gene during the innate immune response. Interestingly, BRD4 activates the expression of NFκB/RelA, a coactivator that binds to BRD4 in a BD-dependent manner. We extend this finding to show that BRD4 also regulates other components of its functional interactome, including the Mediator (Med) coactivator complex and the SWI/SNF-related, matrix-associated, actin-dependent regulator of chromatin (SMARC) subunits. To provide further insight into mechanisms for BRD4 in RSV expression, we mapped 7,845 RSV-inducible Tn5 transposase peaks onto the BRD4-dependent gene bodies. These were located in promoters and introns of cytostructural and extracellular matrix (ECM) formation genes. These data indicate that BRD4 mediates the dynamic response of airway epithelial cells to RNA infection by modulating the expression of its coactivators, controlling the expression of host defense mechanisms and remodeling genes through changes in promoter accessibility.

## Introduction

Bromodomain-containing 4 (BRD4) is a member of the bromodomain and extra-terminal domain (BET) family of proteins that plays pleiotropic roles in epigenetic control of inflammation-inducible gene expression (Nowak et al., 2008; Tian et al., 2016), maintenance of cellular identity (Loven et al., 2013), DNA damage signaling (Floyd et al., 2013), chromatin compaction/conformation (Devaiah et al., 2016a), cell cycle regulation, and so on [Reviewed by Devaiah et al. (2016b)].

In this study, we focus on the activity of BRD4 in controlling inducible innate antiviral genomic response. Previous mechanistic studies have shown that BRD4 forms signal-inducible interactions with nuclear factor-κB (Nowak et al., 2008), heat shock factor (Lis et al., 2000), and SMAD3 (Ijaz et al., 2017) to bind silent gene promoters in euchromatin domains. Here, BRD4 functions as a pause–release factor, releasing inactive RNA polymerase II (Pol II) to enter a fully processive mode (Price, 2000). In this context, BRD4 plays pleiotropic roles as an acetylated histone “reader”, a selective histone acetyltransferase (Devaiah et al., 2016a), and a kinase for cyclin-dependent kinase 9 and RNA polymerase II (Devaiah and Singer, 2012). Although BRD4 interactions with the cyclin-dependent kinases in the P-TEFb complex are well established (Jeronimo et al., 2007; Yang et al., 2015), BRD4 interacts with a spectrum of other chromatin-modifying activities that may be functionally important. Recent studies applying native affinity isolation coupled with high-resolution mass spectrometry have substantially expanded the known protein interactions of BRD4 to include RNA polymerases, coactivators, transcription factors, histone acetyltransferases, and actin motors (Zhang et al., 2017; Mann et al., 2021). In this way, BRD4 forms highly dynamic complexes in a stimulus- and cell-type– dependent manner.

BRD4 interacts with acetylated histones and transcription factors through its conserved bromodomain (BD), with transcriptional regulators through its extraterminal domain, and with transcriptional elongation kinases through its carboxyterminal domain (Bisgrove et al., 2007; Rahman et al., 2011; Zou et al., 2014; Mann et al., 2021). The recent development of highly selective small-molecule BRD4 BD inhibitors (Liu et al., 2018) have enabled the ability to probe the role of BRD4 BD interactions in complex biological responses associated with epithelial inflammation, trained immunity, and cell-state changes (Tian et al., 2019a; Tian et al., 2019b; Zhao et al., 2019). These reagents have enabled studies that have placed BRD4 in a central role in host defense mechanisms to RNA viruses. Here, we focus on the role of BRD4 BD interactions in host defense mechanisms to respiratory syncytial virus (RSV). RSV is an enveloped, single-stranded, negative-sense RNA virus of the *human orthopneumovirus* genus of the Pneuomoviridae family that represents the most common cause of pediatric hospitalization in children less than 5 years of age (Pneumonia Etiology Research for Child Health (PERCH) Study Group, 2019). After inoculation into the nasopharynx, RSV fuses with ciliated epithelial cells and replicates to high levels, spreading throughout the airways to the conductive and lower airway cells involved in gas exchange. As part of a stereotypic innate response viral RNA patterns, BRD4 is activated downstream of the toll-like receptor (TLR) and retinoic acid–inducible gene (RIG-I) pattern recognition pathways (Tian et al., 2017b; Tian et al., 2019b). Consequently, productive viral infection in small airway epithelial cells induces the rapid secretion of cytokines (Zhang et al., 2001), interferons [IFNs, (Liu et al., 2007)], exosomes (Zhao et al., 2017), and damage-associated patterns (Hosakote et al., 2016) that mediate mucous production, leukocytic inflammation, and hypoxia (Bennett et al., 2007; Lay et al., 2016). Through its actions, BRD4 shapes the nature, timing, and magnitude of inducible epithelial cytokine responses, playing an important role in mediating disease and activating adaptive immunity.

Using high-resolution genome-wide studies of chromatin accessibility (ATAC-Seq) and RNA-Seq studies, we recently discovered that RSV infection reprograms gene loci encoding cytokines, growth factors, and extracellular matrix remodeling genes, resulting in their highly dynamic expression (Xu et al., 2020). The role of BRD4 in coordinating the innate response network has not been systematically elucidated. In this study, we study the effect of disrupting BRD4 BD1 and BD2 interactions using highly selective small-molecule BRD4 inhibitors in the RSV-induced gene regulatory response. We present evidence that BRD4 is autoregulated during the innate immune response. Interestingly, BRD4 mediates RSV-inducible expression of its major interacting coactivators, including the transcription factor NFκB/RelA, the Mediator (MED) coactivator complex, and subunits of the SWI/SNF-related, matrix-associated, actin-dependent regulator of chromatin (SMARC). A subset of these genes exhibit enhanced accessibility of their proximal promoters during RSV infection. These data provide insights into the new mechanistic role of BRD4 in RNA virus infections.

## Materials and Methods

### Reagents and Chemicals

The BRD4 selective BD competitive inhibitor, ZL0454, was synthesized as previously described (Liu et al., 2018; Tian et al., 2019b) and used at purity > 99%. ZL0454 was resuspended in DMSO and added to the culture medium at indicated concentrations. Solvent alone (DMSO) was used in non–BRD4 inhibitor-treated conditions.

### Cell Culture and Virus Preparation

Human small airway epithelial cells (HSAECs) immortalized with human telomerase/CDK4 were obtained commercially (ATCC). These are non-oncogenic, telomerase-immortalized cells grown in the SAGM small airway epithelial cell growth medium (Lonza, cc-3118) in a humidified atmosphere of 5% CO_2_. Cells were infected at confluence >90% for profiling.

The human RSV long strain was obtained from ATCC, grown in Hep-2 cells, and prepared as described (Ueba, 1978). The viral titer of purified RSV pools varied from 8 to 9 log PFU/ml, determined by a methylcellulose plaque assay (Xu et al., 2020). Viral pools were aliquoted and quick-frozen on dry ice–ethanol and stored at −70°C until they were used.

### RNA Isolation and Quantitative Real-Time PCR (Q-RT-PCR)

Cells were harvested for RNA isolation using the RNeasy kit with on-column DNase digestion (Qiagen). The synthesis of complementary DNAs (cDNAs) was performed using the First Strand cDNA Synthesis Kit (Thermo Scientific). qRT-PCR assays were used for validation using custom primers and SYBR Green Master mix (Bio-Rad) or TaqMan primers and TaqMan Fast Advanced Master mix (Thermo Scientific) as described previously (Xu et al., 2020). The sequences of the SYBR Green PCR primers are *BRD4* Forward (F): 5′- AAA​GAA​GCG​CTT​GGA​AAA​CA; *BRD4* Reverse (R): 5-CGG​TTT​CTT​CTG​TGG​GTA​GC; *PPIA* F 5′-TTC​ATC​TGC​ACT​GCC​AAG​AC′, *PPIA* R 5′-TCG​AGT​TGT​CCA​CAG​TCA​GC; *RELA* F: 5′-CTT​CCA​AGA​AGA​GCA​GCG​TG; *RELA* R: 5′-. CTT​CCA​AGA​AGA​GCA​GCG​TG; *IFNL* F” 5′-: GGA​GTT​GCA​GCT​CTC​CTG​TC; and *IFNL* R 5′- CAG​CGG​ACT​CCT​TTT​TGG​GG. Data are normalized to internal control and presented as fold change using the ΔΔ Ct method.

### RNA-Next Generation Sequencing

Total RNA was isolated using the RNeasy kit with on-column DNase I digestion (Qiagen). RNA was quantified (Nanodrop), and its was integrity verified (Agilent) prior to sequencing. Libraries were produced using the TruSeq Stranded mRNA library kit (Illumina). The 12 separate RNA samples (*N* = 4 each for HSAEC control, RSV, and RSV + ZL0454 24 h of RSV treatment) were bar-coded and subjected to Illumina HiSeq 2000 paired-end sequencing. Quality control was performed using FastQC. Trimmomatic was used to remove sequencing adapters. Trimmed paired-end reads were aligned against human genome hg38 using Salmon. Mapped paired-end reads for both genes and transcripts (isoforms) were counted in each sample using RSEM and expressed as transcripts per million (TPM), the recommended metric for transcript quantification (Patro et al., 2017). Contrasts were compared for RSV or BRD4 inhibitor treatment conditions using DESeq2 (Love et al., 2014).

### ATAC-Seq Data Analysis

ATAC-Seq Fastq files were (Xu et al., 2020) analyzed for read coverage, and PCR duplication and number of mapped reads were determined using FastQC. Adapter removal was performed using TrimGalore (Kreuger, 2020). Alignment was performed using the RSubread package (Liao et al., 2019) to the GRCh38.p13 (hg38) genome assembly (NCBI). Peak calling was performed using Genrich for ATAC-Seq (Gaspar, 2019), using commands to remove mitochondrial sequences, remove PCR duplicate reads, and mask blacklisted sequences. Statistical comparisons were conducted using DESeq2 in the DiffBind package (Ross-Innes et al., 2012). Functional annotation was performed using ReactomePA (Yu et al., 2014). Functional gene enrichment analysis was performed using ClueGO v 2.5.8 plug-in (Bindea et al., 2009) in Cystoscope 3.2 (Shannon et al., 2003).

### Protein Lysate Preparation and Western Blot

HSAECs were trypsinized, pelleted, and washed twice with cold phosphate-buffered saline (PBS). Cell pellets were then lysed in cold low ionic strength buffer (50 mM NaCl, 1% IGEPAL, 10%Glycerol, 10 mM HEPES, pH7.4) with freshly added proteinase inhibitor cocktail (Sigma), DTT and PMSF (Sigma). 10 µl of the cell lysate was removed for protein measurement (DC protein assay kit, Bio-Rad). 2xSDS sample buffer was added. The lysates were sonicated thrice for 15 s (Branson150), centrifuged at 10000 rpm for 20 min, and supernatants were heated at 95^o^C for 5 min. Proteins were resolved using Criterion TGX 4-15% precast gel and transferred to a nitrocellulose membrane using the Bio-Rad Trans-Blot Turbo transfer system. Sources of primary antibodies and dilutions used were anti-RelA: Cell Signaling 8242S, 1:1000; anti-BRD4: Cell Signaling 13440, 1:1000; and anti-GAPDH Abcam ab59164, 1:2000.

### *In Situ* Proximity Ligation Assay

Control, RSV-infected, and RSV + ZL0454–treated HSAECs were fixed with 4% paraformaldehyde (10 min), permeablized with 0.1% Triton X-100 (10 min), and blocked with 5% goat serum in PBS (2 h). The slides were then subjected to PLA using the Duo-link PLAkit from O-Link Bioscience (Uppsala, Sweden) according to the manufacturer’s instructions. Nuclei were counterstained with DAPI, and the PLA signals were visualized using a Nikon A1RS confocal microscope at 63X magnification.

### Statistical Analysis

Statistical analysis of the RNA-Seq data was conducted using the DESeq2 R package (version 1.32.0) (Love et al., 2014). Contrasts with padj <0.05 of log2 fold change (log2FC) by DESeq2 were considered significant. Data are presented as log2(fold change) using a 25–75% interquartile range. Adjusted pvalues for the RSV vs RSV + BRD4 inhibitor contrast are provided in the figure legends. qRT-PCR was compared using ANOVA with Tukey’s post hoc comparisons test. Gene Ontology analysis was performed using Reactome.org (Jassal et al., 2020). Pathway analysis was performed using disease ontology semantic and enrichment analysis (version 3.18.0) (Yu et al., 2014). Data are plotted as 25–75% of the interquartile range.

## Results

Innate signaling in small airway epithelial cells located in the bronchiolar epithelium plays a major role in the pathogenesis of RSV lower respiratory tract infection (LRTI). This conclusion comes from studies where inhibition of NFκB/RelA signaling in bronchiolar small airway cells blocks RSV-induced inflammation and airway obstruction *in vivo* (Tian et al., 2018b). This epithelial subtype plays an important role in the immunopathogenesis of LRTI because these cells are programmed to produce mucin- and T-helper-2 lymphocyte-activating cytokines that mediate the disease (Zhao et al., 2017).

HSAECs are a well-established *in vitro* model of small airway epithelial cells that induce similar global genomic and proteomic responses to RSV as that of primary small airway cells *in vitro* and that of lower airways *in vivo* (Zhang et al., 2001; Zhao et al., 2017). To elucidate the role of BRD4 in the HSAEC antiviral response, we first compared the effects of two highly selective BRD4 inhibitors (Liu et al., 2018; Liu et al., 2020) on RSV-induced cytokine expression. ZL0454 binds with nanomolar affinity to both bromodomains (BDs) of BRD4 with a 30-fold higher affinity than that of other BET family members. ZL0516 preferentially binds to BRD4 BD1 with ∼8.5 times lower IC_50_ value than to BRD4 BD2 (Liu et al., 2020).

**FIGURE 1:**
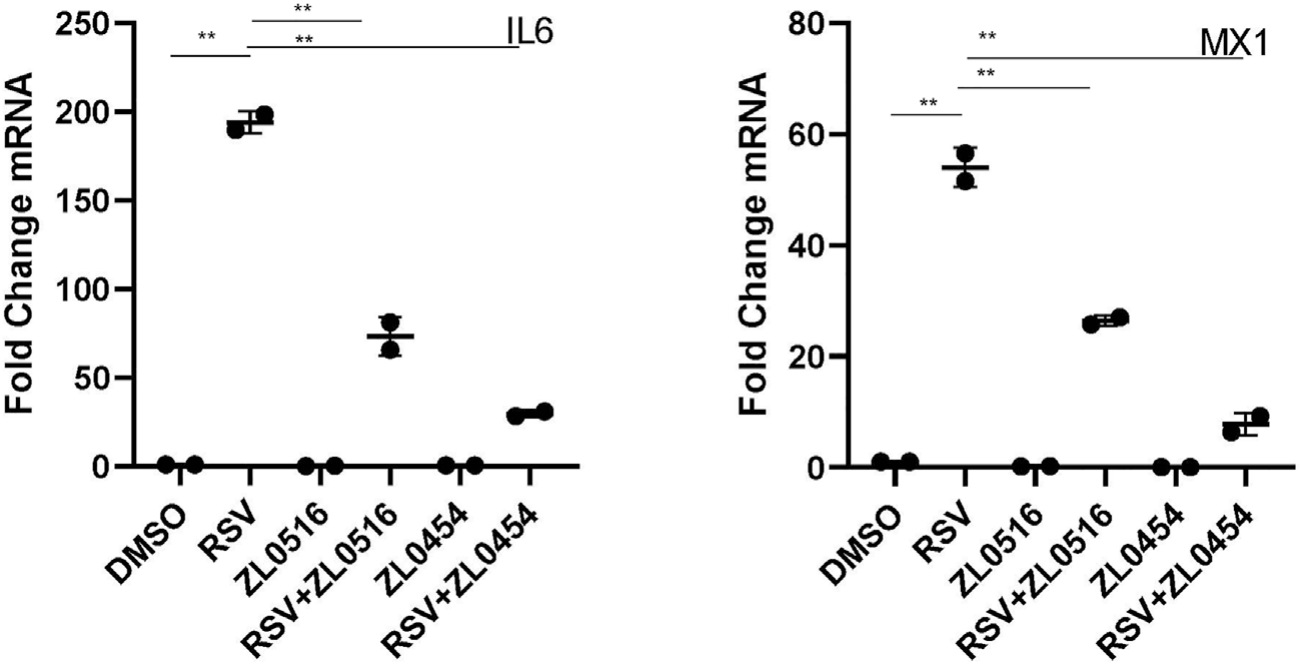
Effect of BRD4-selective small-molecule inhibitors on RSV-induced cytokines. qRT-PCR was performed in HSAECs treated in the absence or presence of 10 μM concentrations of indicated BRD4 inhibitors. HSAECs were infected with purified RSV (MOI = 1) for 24 h. Fold change mRNA relative to PPIA is plotted for *IL-6* **(left)** and *MX1* **(right)**. *p* < 0.01 in ANOVA across treatment groups; ** and *p* < 0.01 in pairwise comparison.

Wild-type (WT) HSAECs were infected with sucrose-purified RSV [multiplicity of infection (MOI) = 1] in the presence of DMSO (solvent control), ZL0454 (10 μM), or ZL0516 (10 μM). 24 h later, the expression of two known BRD4-dependent genes, interleukin 6 (*IL-6*) and MX dynamin-like GTPase 1 (*MX1)*, was measured by qRT-PCR. RSV induced a ∼190-fold increase in *IL-6* expression; both ZL0454 and ZL0516 significantly inhibited its expression, with the inhibition of ZL0454 being consistently greater (Figure 1). Similar inhibitory effects were observed for the ∼55-fold induction of *MX1* (Figure 1). Because ZL0454 showed consistently greater inhibitory activity, is more potent than the prototypical inhibitors JQ1 and RVX20 (Tian et al., 2019b), and exhibits no detectable cellular toxicity(Tian et al., 2019b), we selected ZL0454 for subsequent RNA-seq experiments.

### Exploratory Data Analysis

**FIGURE 2:**
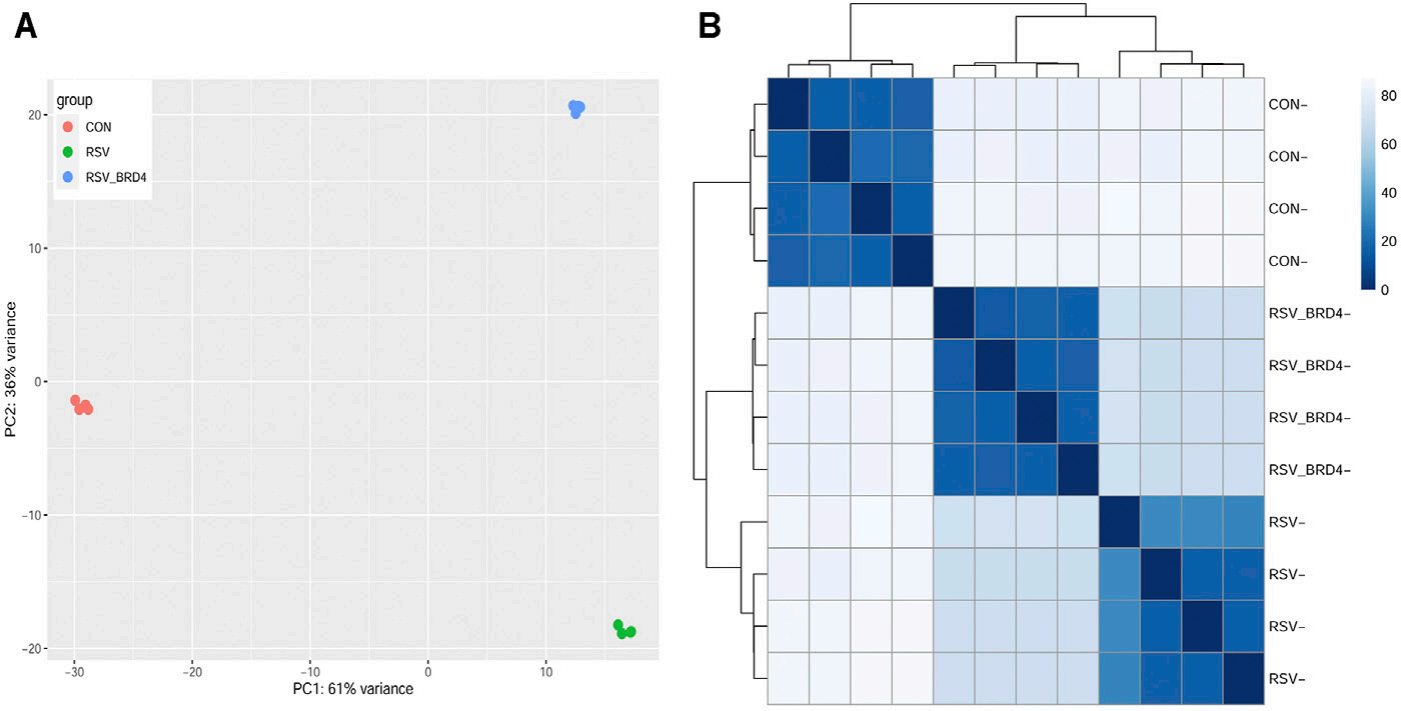
Sample QC. **(A)** Principal component analysis. X axis, first principal component; Y axis, second principal component. Note that the first principal component separates the RSV (±BRD4 inhibitor) from the CON-treated samples and represents 61% of the variability in the experiment. The second dimension separates the RSV vs RSV + BRD4 inhibitor treatment. **(B)** Correlation analysis of log-transformed RNA-Seq quantitation of the experimental samples. Samples were subjected to hierarchical clustering, with distance indicated in the dendrogram. Note that the individual CON, RSV-BRD4 inhibitor, and RSV-infected samples cluster with other replicates receiving the sample treatment and separate from other treatments.

Four replicates of control, RSV-infected or RSV + BRD4 inhibitor (ZL0454)–treated HSAECs were subjected to short read Illumina HiSeq 2000 paired-end sequencing (Methods). To examine whether the RNA expression patterns were reproducible and similar with respect to biological replicates, principal component analysis (PCA) was performed. We observed that the control (CON), RSV-infected, and RSV- ZL0454–treated samples were widely separated in the PCA. The two principal component dimensions accounted for 97% of the sample variability, indicating that the data represented a robust effect of RSV infection and BRD4 inhibitor treatment (Figure 2A). Moreover, the four experimental replicates were virtually superimposed, indicating a highly reproducible dataset. The first principal component separated the RSV-infected cells (±BRD4 inhibitor) vs controls (CON). By contrast, the second PCA component separated the RSV-infected cells with DMSO vs those with BRD4 treatment. Separately, the distance matrix of the log-transformed TPM quantitation was subjected to hierarchical clustering (Figure 2B). In a manner consistent with the PCA result, control (CON), RSV-BRD4 inhibitor, and RSV-infected sample cluster with other replicates received the sample treatment and were separated from other treatments. These data indicate that the RNA-Seq captures reproducible information about the effects of RSV and that of the inhibitor, and that the inhibitor does not revert the cell state back to that of the control.

### Effect of Bromodomain-Containing Protein 4 Inhibitor on Respiratory Syncytial Virus Expression

**FIGURE 3:**
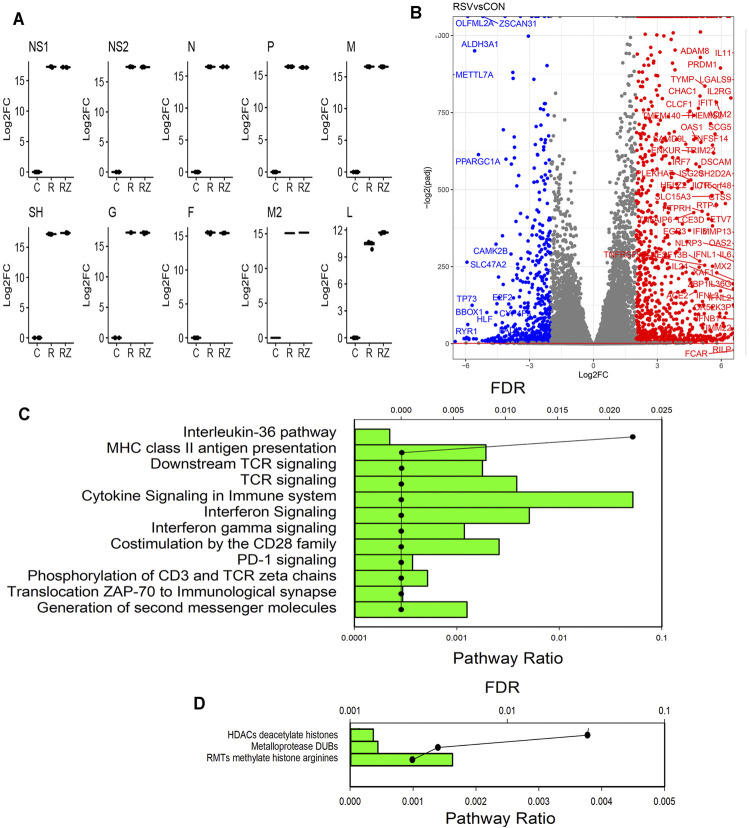
RSV-induced gene regulatory network. **(A)** Quantitation of RSV transcripts. Log2 fold change (log2FC) values of short-read RNA-Seq are plotted by quartile (25–75%) box plots. The horizontal line is the mean value. For each transcript group, comparisons are significantly different for the RSV-infected vs mock-infected (CON) contrast. DZD2, RNA-dependent RNA polymerase; F, fusion protein; G, glycoprotein; M, matrix protein; N, nucleoprotein; NS, nonstructural protein; P, phosphoprotein; and SH, small hydrophobic protein. **(B)** Volcano plot of differentially expressed genes (DEGs) in CON vs RSV-infected cells. X axis, log2fold change of transcripts/million (TPM). A positive value in red indicates that the gene is upregulated by RSV infection. Y axis, −log10(adjusted *p* value using Benjamini-Hochberg, padj). Blue are genes downregulated by RSV (log2FC > 2). Red are genes upregulated. **(C)** Genome Ontology (GO) enrichment of RSV-upregulated genes. Genes with 4-fold change in TPM (RSV *vs* CON) and adjusted *p*-value <0.01 were analyzed for pathway enrichment (reactome.org). For each gene set, the fraction of genes represented in the pathway and the significance (false discovery rate, FDR) are plotted. Shown are the pathways with FDR <0.05. **(D)** GO enrichment of RSV-downregulated genes. Genes with <-4-fold change in TPM (RSV *vs* CON) and adjusted *p*-value of <0.01 were analyzed for pathway enrichment as given above.

RSV is sequentially transcribed into 10 polyadenylated mRNA species from a 3′ to 5′ direction by its RNA-dependent RNA polymerase in the order 3’ (leader) -NS1-NS2-N-P-M-SH-G-F-M2- DZD2-(trailer) 5′ using a “gene start”– “gene stop” mechanism (Collins and Graham, 2008). Our previous study has shown no effect of BRD4 silencing or inhibition on RSV replication *in vitro* (Tian et al., 2017b). To confirm, we quantitated individual mRNA species (Figure 3A). Importantly, we found that the IRF3/IFN antagonists, NS1 and NS2, were expressed at indistinguishable levels. Consistent with the 3′-5′ gradient of expression, the abundance of NS1 (in TPM) was 75-fold greater than that of low-abundance RNA polymerase (L) transcript (not shown). Moreover, we found that RSV transcription was essentially unchanged in the BRD4 inhibitor–treated cells, and that, in fact, SH and L expression were elevated over that of RSV infection alone (Figure 3A). Together, these data indicated that BRD4 inhibition had little overall effect on RSV transcription in this *in vitro* model.

### Respiratory Syncytial Virus Regulated Gene Networks

RSV replicates in primary human airway epithelial cells, inducing a well-established, time-dependent global genomic response, including both gene activation and repression (Zhang et al., 2001; Xu et al., 2020; Xu et al., 2021a). Consistent with this earlier study, our data indicate that RSV induces dramatic changes in the expression of 11,038 genes (5620 upregulated and 5418 downregulated). A stringent filter of differentially expressed genes is illustrated by the volcano plot. In this analysis, the statistical significance of the change in expression of each gene in the dataset [−log_10_(p_adj_)] is plotted versus the relative change in expression (expressed in log_2_ fold change, Figure 3B). RSV induces substantially greater number of genes than are inhibited. The most highly upregulated and downregulated gene sets ( |log_2_FC| > 4) were separately analyzed by Gene Ontology (GO) analysis, where pathway enrichment is represented statistically by enrichment relative to the human genome (FDR) and by the fraction of genes in the dataset that map to any given pathway (pathway ratio). The GO analysis of the upregulated genes identifies the enrichment of well-established RSV-induced pathways, including T-cell receptor signaling, innate responses, IL-10 signaling, and IL-36 signaling (Figure 3C). By contrast, GO analysis of downregulated genes identified gene mapping to HDAC, metalloproteinases, and arginine methylation pathways (Figure 3D). We conclude that the RSV-inducible gene expression programs in these data are highly representative of known RSV programs.

### Bromodomain-Containing Protein 4 Inhibitor–Modulated Gene Regulatory Networks

**FIGURE 4:**
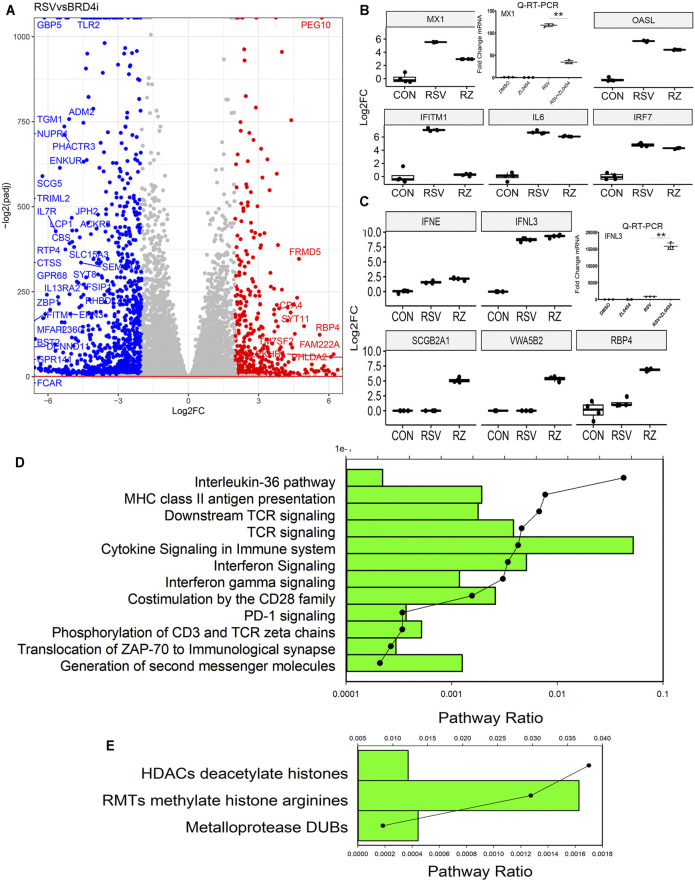
BRD4-dependent gene network. **(A)** Volcano plot of BRD4-dependent genes comparing RSV vs RSV + BRD4 inhibitor–treated cells. Genes downregulated by BRD4 inhibitor are plotted in blue and those upregulated by BRD4 inhibitor are shown in red. **(B)** RSV-inducible interferon stimulatory genes dependent on BRD4. First graph is RNA-Seq data (log_2_FC) for the *MX1* gene. Adjusted pValue for RSV vs RSV + ZL0454 = 1.12E-15. Right graph is qRT-PCR of an independent experiment. *MX1* expression is shown as fold change over mock-infected controls. **, *p* < 0.01 by post hoc Tukey’s comparison test. Remainder of graphs are RNA-Seq log_2_FC presented as 25-75% interquartile graphs. Adjusted *p* values for RSV *vs* RSV + ZL0454 contrast are *IFITM1* = 7.64E-47, *OASL* = 7.97E-34; and *IRF7* = 0.00038. **(C)** RSV-inducible genes repressed by BRD4. RNA-Seq of *IFNL3*, where ZL0454 increases the RSV-induced expression. Adjusted *p* value of the RSV vs RSV + ZL0454 contrast is = 0.0019. Right graph, validation of *IFNL* mRNA expression by independent qRT-PCR. **, *p* < 0.001. Adjusted *p* Values for RSV *vs* RSV + ZL0454 contrast in DESeq2 are as follows: *SCGB2A1* = 7.48E-7; *VWASB2* = 1.147E-7; *IFNE p* = 0.00041; and *RBP4* = 7.73E-38. **(D)** Genome Ontology (GO) enrichment of BRD4-dependent RSV-upregulated genes. Genes with 4-fold log_2_FC (RSV *vs* RSV + ZL0454) and adjusted *p*-value <0.01 were analyzed for pathway enrichment (reactome.org). For each gene set, the fraction of genes represented in the pathway and the significance (false discovery rate, FDR) are plotted. Shown are the pathways with FDR <0.05. **(E)** GO enrichment of RSV-upregulated genes repressed by BRD4. Genes with <-4-fold change in TPM (RSV *vs* RSV + ZL0454 ) and adjusted *p*-value of <0.01 were analyzed for pathway enrichment as given above

We next focused on the gene regulatory network affected by ZL0454. Contrasts of gene expression were analyzed for the effect of BRD4 controlling for multiple hypothesis testing in DESeq2. 10,559 genes were differentially expressed with an adjusted *p* value <0.01. The differentially expressed genes between RSV + DMSO vs RSV + ZL0454 were plotted by the adjusted statistical significance of the change (−log_10_(p_adj_)) versus the relative change in expression (expressed in log_2_FC of the TPM, Figure 4A). A much greater number of genes are downregulated by the BRD4 inhibitor (indicated in blue, Figure 4A) than those upregulated (indicated in red, Figure 4A). To visualize the expression patterns, the normalized expression values of index genes in the “Cytokine Signaling in the Immune System” were retrieved and analyzed by comparing the log2-transformed fold change (log2FC). The index gene in this group is the RSV- and IFN-inducible MX dynamin-like GTPase 1 (*MX1)*, shown in Figure 4B. In the RNA-Seq dataset, the expression of *MX1* is dramatically induced by ∼5.8 log units by RSV and significantly inhibited by ZL0454 (Figure 4B, left graph). To validate this pattern, wild-type HSAECs were separately treated with solvent (DMSO) or ZL0454 and then mock- or RSV infected. Total RNA was extracted, and qRT-PCR was performed for *MX1*. RSV potently upregulated *MX1* expression in solvent-treated HSAECs by ∼120-fold, but this induction was substantially reduced in ZL0454-treated cells (Figure 4B, right graph), exactly reproducing the pattern in the RNA-Seq data.

In a similar manner, other index genes in the “cytokine signaling in the immune system”pathway, including IFN- induced transmembrane protein 1 *(IFITMI),* 2′-5′-oligoadenylate synthetase-like *(OALS)* and interferon response factor 7 (*IRF7*) genes were highly upregulated by RSV and inhibited by ZL0454 (Figure 4B). Genes paradoxically increased by BRD4 inhibition were selected by rank order of fold change in TPM RSV vs RSV + ZL0454 and similarly analyzed. In contrast to the behavior of *MX1* and *OASL*, IFN lambda (*IFNL3*) was induced by RSV and further increased by BRD4 inhibition (Figure 4C, left graph). This pattern of behavior was independently validated by qRT-PCR (Figure 4C, right graph). Other genes showing paradoxical induction by the BRD4 inhibitor included secretoglobin family 2A member 1 (*SCGB2A1*), Von Willebrand factor A domain (V*WASB2*), and retinol binding protein 4 (*RBP4*) (Figure 4C). Although the differences are highly consistent and statistically significant, we note that the abundance of these transcripts is quite low in comparison to those inhibited by BRD4. To further understand the functional activities of the BRD4-activated and BRD4-repressed genes, GO analysis was performed for each group separately. 273 genes were downregulated by 4- log_2_FC in the BRD4 inhibitor group, and 78 genes were upregulated by 4 log_2_FC in the BRD4 inhibitor relative to that of RSV infection alone. We noted a striking similarity of the functional pathways of the BRD4-dependent genes as that of the RSV-dependent genes, although the pathway enrichments and statistical confidence were not as high (Figures 4C,D). We conclude from these data that BRD4 is both an activator and repressor of IFN-stimulated genes (ISGs) and growth factor–responsive genes during the evolution of the antiviral response.

**FIGURE 5:**
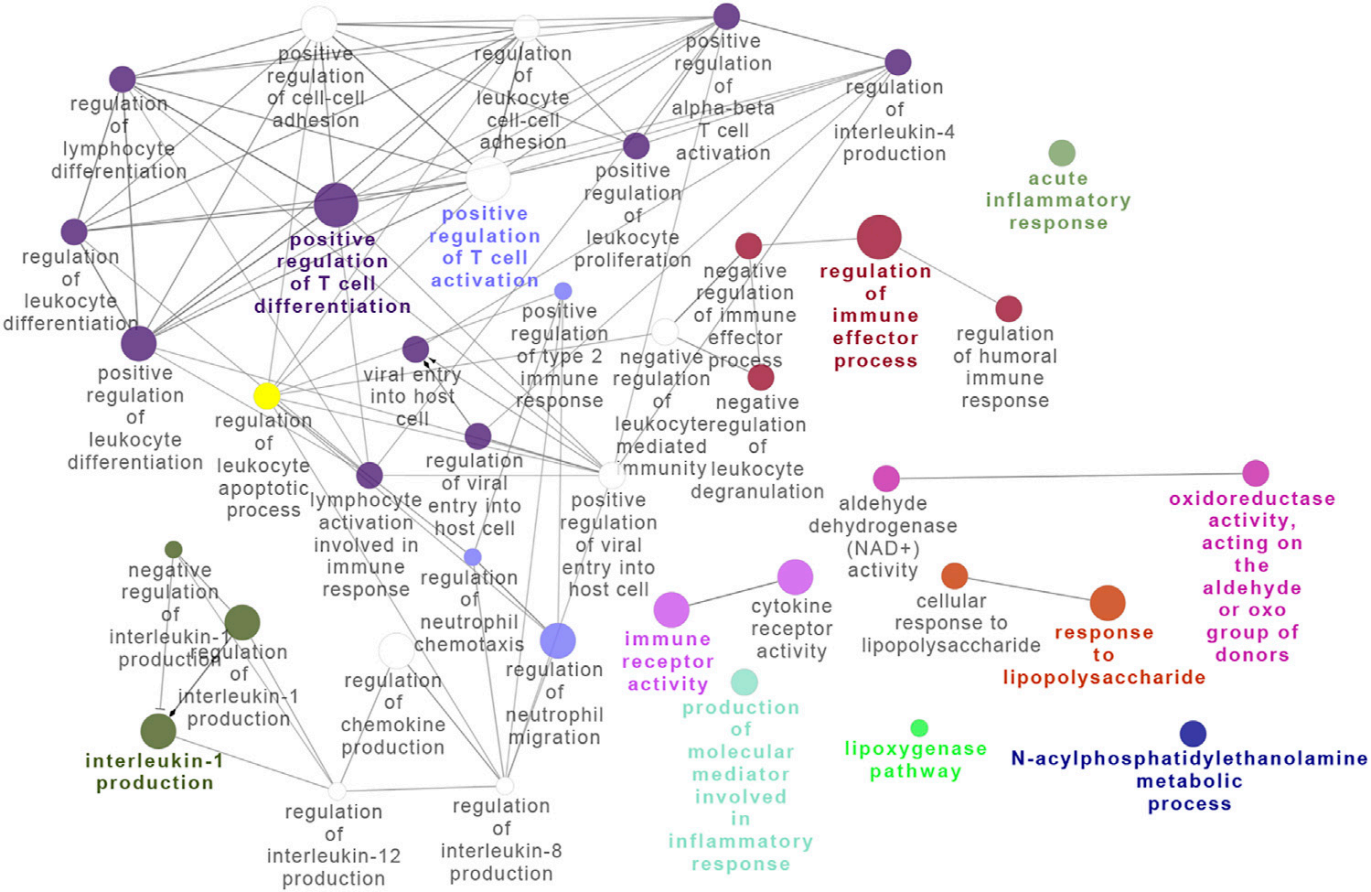
Integrated network analysis of BRD4-dependent genes. Gene pathway interaction map of BRD4-dependent genes. Note the striking enrichment of T-cell differentiation and activation of immune effector processes.

To further examine the BRD4-dependent gene regulatory network in RSV infection, we subjected the BRD4-dependent genes to integrated GO analysis. Here, densely connected networks of genes controlling the regulation of T-cell differentiation, immune effector processes, and interleukin expression were identified (Figure 5), consistent with the anti-inflammatory properties of BRD4 inhibitors in RSV infection *in vivo* (Tian et al., 2018a; Tian et al., 2019b; Zhao et al., 2019). A network analysis incorporating disease and protein–protein interaction information was also conducted using DOSE (data not shown). This analysis linked the BRD4-regulated network with epithelial carcinoma–driven states, consistent with its role in oncogenesis. This analysis also identified BRD4-regulated chromatin-modifying genes, including cyclin B1 (*CCNB1*), enhancer Of zeste 2 polycomb repressive complex 2 subunit (*EZH2*), GATA-binding protein 3 (*GATA3*), and Forkhead Box A1.

(FOXA1)These findings suggested that BRD4 may regulate the expression of other chromatin modifiers.

### Bromodomain-Containing Protein 4 is Autoregulated and Controls NFκB/RelA Expression

**FIGURE 6:**
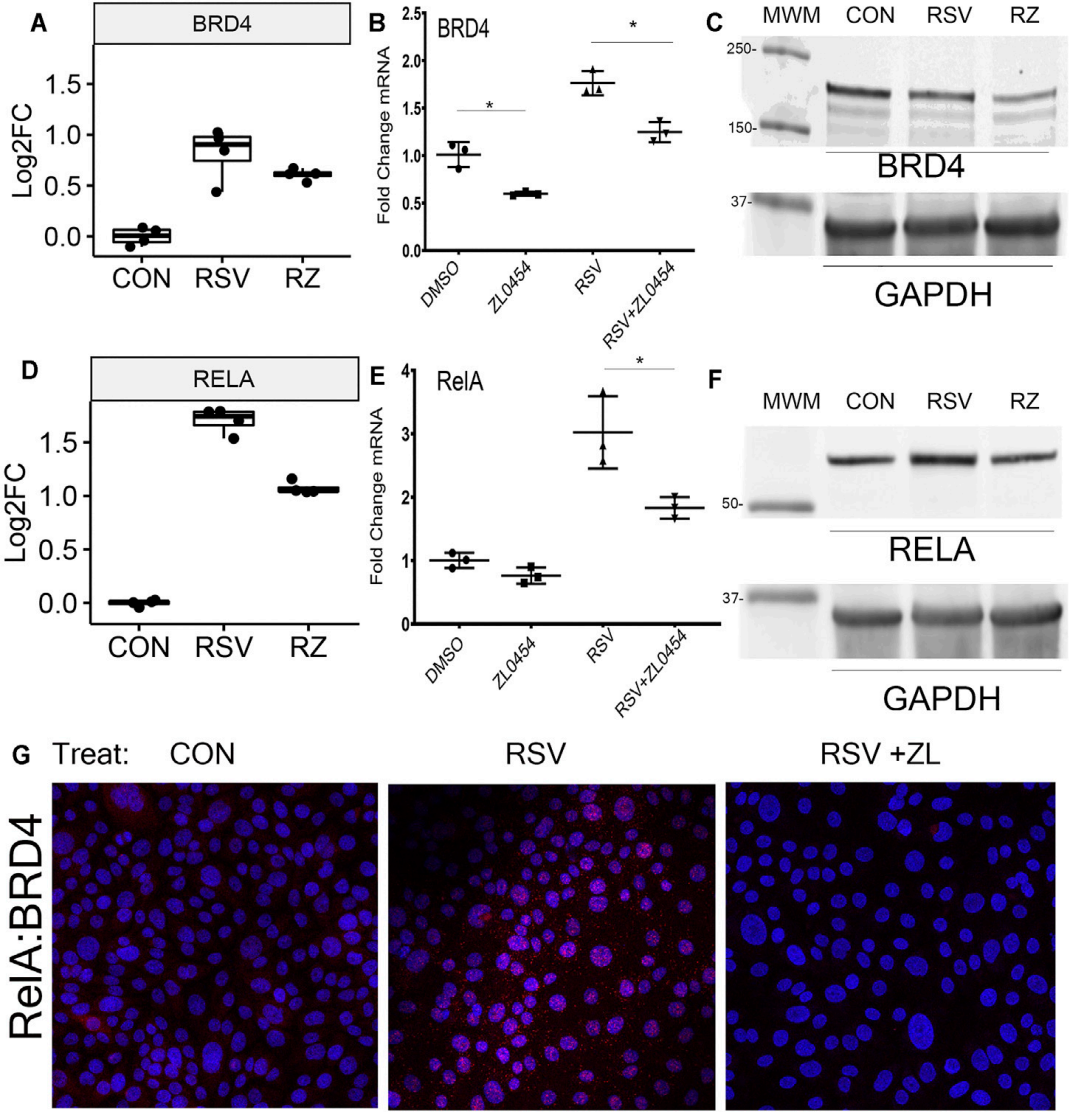
BRD4 autoregulation. **(A)** BRD4 mRNA expression. Shown are log2FC counts from RNA-Seq plotted as 25-7% interquartile ranges. Adjusted *p-*value for DESeq2 contrast RSV vs RSV + ZL0454 is 0.043. **(B)** Validation in independent experiments by qRT-PCR. *BRD4* mRNA is expressed as fold change over mock-infected controls. Note the basal and RSV-induced induction of *BRD4* mRNA is blocked by the BRD4 inhibitor. *p < 0.05 post hoc Tukey’s comparison test. **(C)** Western immunoblot. Whole cell lysates were fractionated by SDS-PAGE and blotted for BRD4. GAPDH staining is the internal control. MWM, molecular weight markers (in kDa). **(D)** *RELA* mRNA expression in log2FC. Adjusted *p-*values for the RSV vs RSV + ZL0454 contrast is 6.07E-23. **(E)** *RELA* mRNA expression by qRT-PCR. *p < 0.05 post hoc Tukey’s comparison test. **(F)** NFκB/RelA expression. Western blot of whole cell extracts. GAPDH staining is the internal control. **(G)** Proximity ligation assay. RelA–BRD4 interaction was quantified using PLA. BRD4–RelA complexes were quantified in the control group(Con), RSV-infected, or RSV + ZL–treated HSAECs. Red dots are BRD4-RelA complexes and nuclei are counterstained with DAPI (Blue). Note that the marked induction of BRD4-RelA complexes by RSV is blocked by ZL0454 treatment.

Informed by the findings that BRD4 regulates many chromatin modifiers and our previous findings that BRD4 protein and mRNA expression are induced by RSV infection (Tian et al., 2017b), we explored the effects of ZL0454 on RSV-induced BRD4 expression. Strikingly, a ∼0.8 log2FC increase in BRD4 mRNA produced by RSV infection is reduced by ZL0454 treatment (Figure 6A), a finding independently validated by qRT-PCR, where the effect of ZL0454 on basal and RSV-induced expression is observed (Figure 6B). In Western blot, RSV slightly reduced the steady-state abundance of ∼170 kDa BRD4 protein, suggesting RSV induces the turnover of the activated protein. Consistently with that of the transcript abundance, BRD4 protein abundance was inhibited by ZL0454 treatment (Figure 6C).

The association of BRD4 with the innate inducible transcription factor, NFκB, is a major component of the antiviral transcriptional elongation response to RSV (Brasier et al., 2011; Tian et al., 2018b; Mann et al., 2021). Although RSV regulates NFκB at a posttranscriptional level by inducing its nuclear–cytoplasmic translocation (Garofalo et al., 1996; Jamaluddin et al., 1998; Tian et al., 2002; Choudhary et al., 2005), RSV infection also induces the synthesis of *NFκB/RelA* mRNA by a largely unknown mechanism (Tian et al., 2018b). Extending these earlier findings, here we find that RSV-induced *RELA* mRNA expression is BRD4-dependent, where *RELA* mRNA abundance increases ∼1.7 log units in response to RSV and is substantially reduced by ZL0454 treatment (Figure 6D). This induction of *RELA* is independently validated by qRT-PCR, where RSV induces a 3-fold change in normalized *RELA* transcripts, an amount reduced to ∼1.8 fold by ZL0454 treatment (Figure 6E). A 1.5-fold induction of steady-state RelA abundance by RSV is confirmed by Western blot, and the levels are reduced to less than the control in response to ZL0454 treatment (Figure 6F). RelA–BRD4 interaction was confirmed in proximity ligation assay (PLA). RSV infection markedly induced the association of BRD4 with RelA in HSAECs, an association blocked by ZL0454 treatment (Figure 6G). These data suggested the very intriguing possibility that the BRD4 interactome was being controlled by BRD4 action.

### Bromodomain-Containing Protein 4 Expression Modulates the Expression of Its Interactome

**FIGURE 7:**
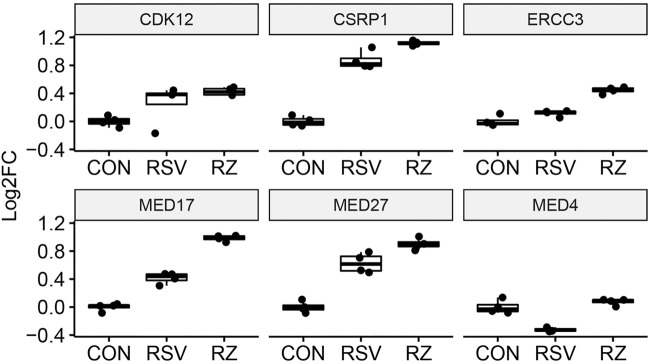
BRD4 modulates the expression of MED. mRNA expression of BRD4-interacting MED complexes. For each, log_2_FC values are plotted as 25-75% interquartile ranges. Adjusted *p-*values of RSV vs RSV + ZL0454 treatment are as follows: *MED4* = 0.000144; *MED27* = 0.00159; *CSRP1* = 0.00102; *MED17* = 2.1E-25; *ERCC3* = 2.08E-8; and *CDK12* = NS.

**FIGURE 8:**
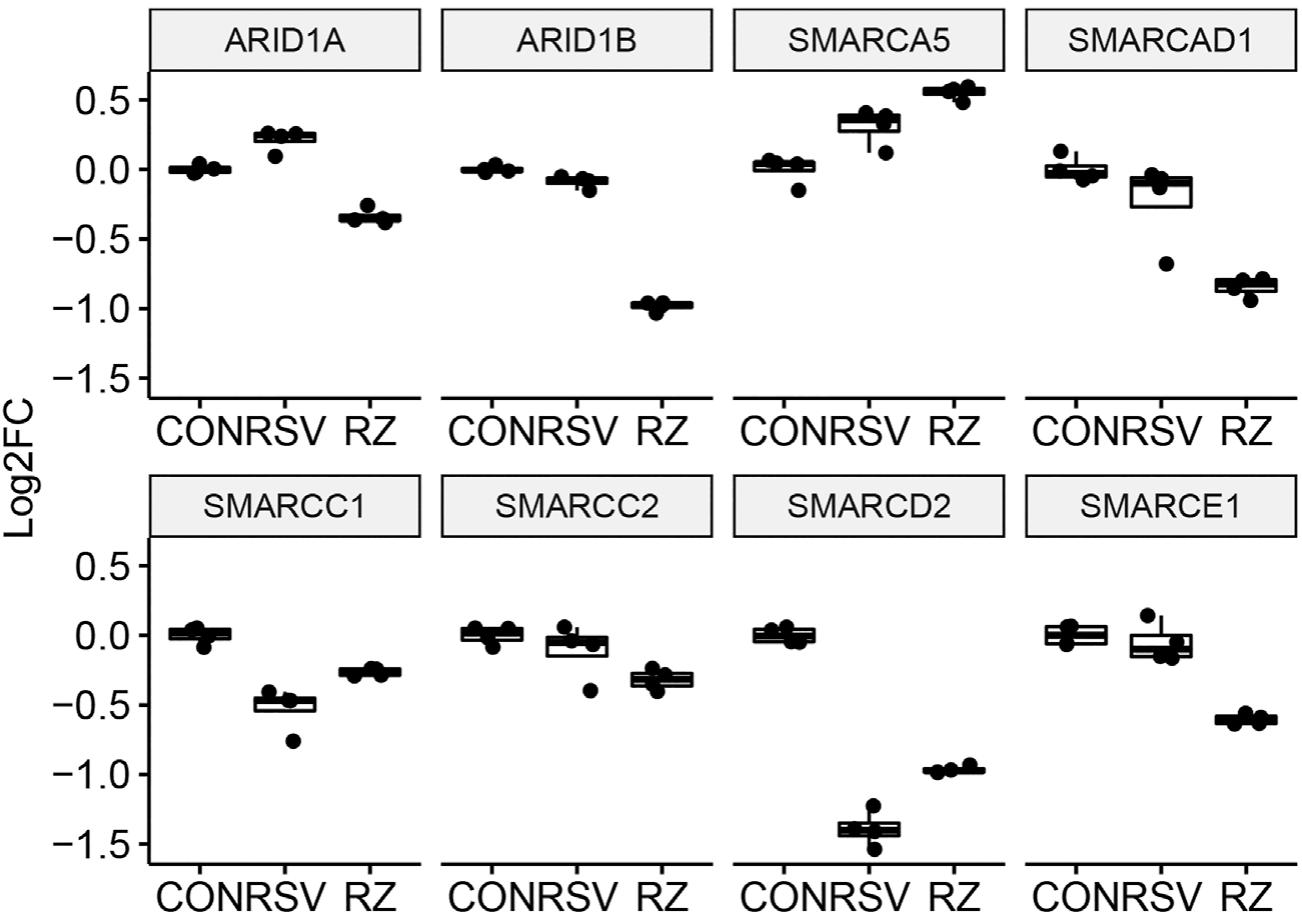
BRD4 modulates the expression of SMARC complexes. mRNA expression of BRD4-interacting SMARC complexes. For each, log_2_FC values are plotted as 25-75% interquartile ranges. Adjusted *p-*values of RSV vs RSV + ZL0454 treatment are as follows: ARI1A = 1.21E-29; ARID1B = 2.82E-98; SMARCE1 = 8.13E-14; SMARCD2 = 8.37E-15; SMARCC2 = 0.024; SMARCA5 = 0.0014; SMARCAD1 = 2.81E-7; and SMARCC1 = 0.000723.

This finding that BRD4 regulates NFκB/RelA expression in the antiviral response raised the question whether BRD4 controls other members of its interactome. Our recent study using native affinity enrichment and parallel accumulation–serial fragmentation mass spectrometry identified >100 BRD4-associated coactivators and transcription factors whose RSV-induced interactions were disrupted by BRD4 BD inhibitors (Mann et al., 2021). Protein–protein interaction network analysis indicated that BRD4 interacts with the Mediator complex and the SWI-SNF family of ATP-dependent chromatin interactors. Mediator (MED) is a multi-subunit adapter complex that mediates long-range enhancer interactions important in steroid receptor– induced gene transcription (Zhang et al., 2005). Intriguingly, BRD4 repressed *MED4*, *MED27*, cysteine- and glycine-rich protein 1 (*CSRP1*), *MED17,and* the ERCC excision repair 3 (*ERCC3*) and cyclin-dependent kinase (*CDK12*) expression (Figure 7). By contrast, BRD4 activates AT-rich interaction domain 1A (*ARID1A*), *ARID1B, SMARC-E1, C2,* and -*D1*. By contrast, BRD4 suppresses *SMARCA5* (Figure 8). These data indicate that BRD4 controls a complex-coordinated regulation of its interactome in the antiviral response.

### A Subset of Bromodomain-Containing Protein 4-dependent Genes Undergo Respiratory Syncytial Virus-Induced Chromatin Remodeling

**FIGURE 9:**
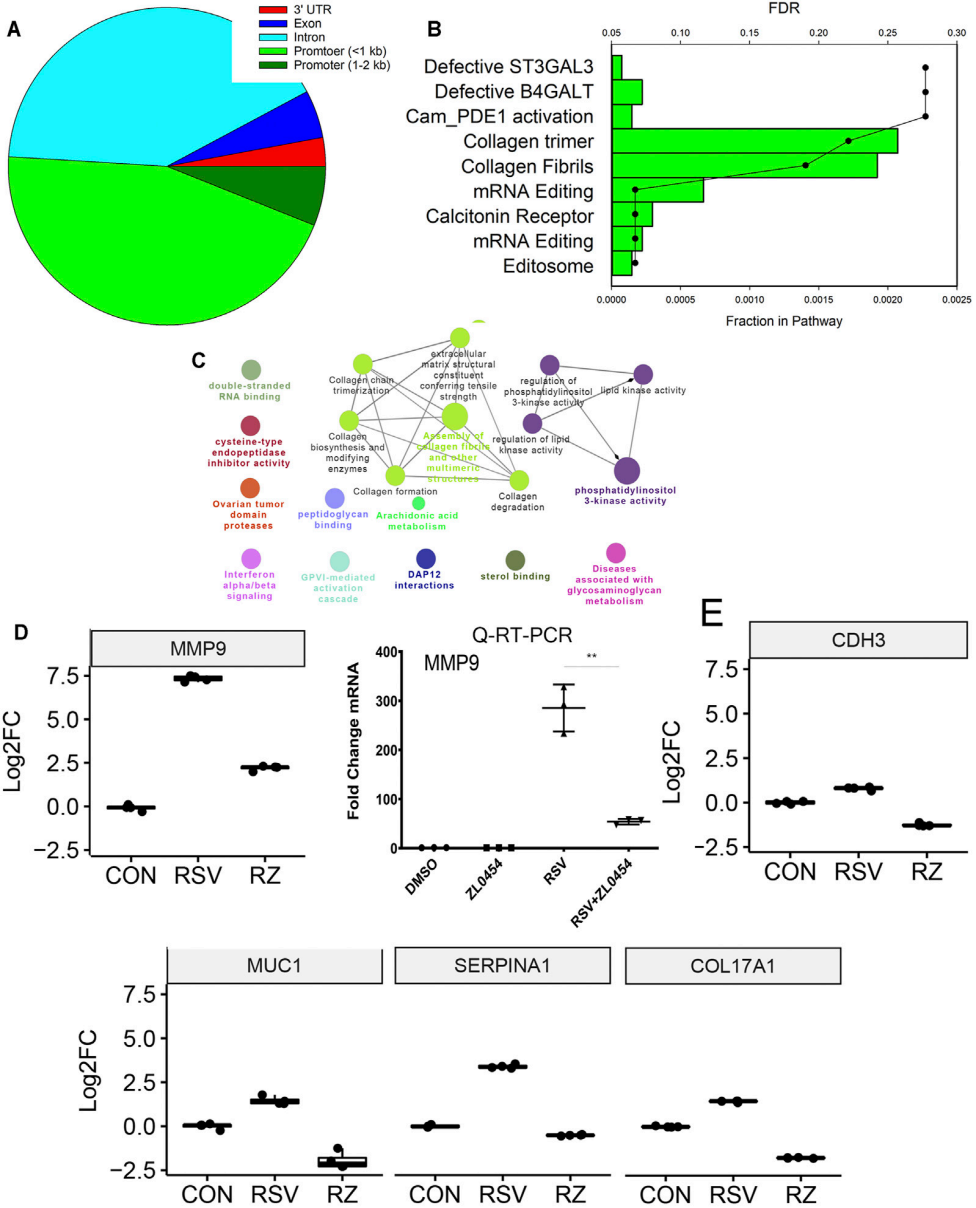
BRD4-dependent genes with RSV-inducible chromatin. 7,845 RSV-inducible Tn5 transposase-accessible genomic cleavage sites were mapped to BRD4-dependent gene bodies. 516 peaks were mapped to BRD4-dependent genes. **(A)** Location of cleavage sites on gene bodies. **(B)** GO enrichment analysis. Bars, fraction of pathway in datasets. Scatterplots are false discovery rate for pathway enrichment. Note the enrichment of collagen trimers and fibril formation. **(C)** Integrated GO enrichment. A major network involved in ECM and collagen is indicated in green. **(D)** MMP9 mRNA expression in RNA-Seq is validated by qRT-PCR. Left, log2FC from RNA-Seq. Adjusted *p-*values is < E-100. Right, fold change mRNA by qRT-PCR. **(E)** mRNA expression of other ECM and structural index genes are plotted as 25–75% interquartile ranges. Adjusted *p-*values of RSV vs RSV + ZL0454 treatment are: COL17A = E < -100; MUC1 = 1.4E-76; SERPINA1 = E < -100; and CDH3 = 3.33E-236.

Earlier, we reported a genome-wide analysis of RSV-induced chromatin remodeling using Tn5 transposase cleavage and next generation sequencing, for example, ATAC-Seq (Xu et al., 2020). Here, we identified functionally related clusters of genes controlling ECM remodeling and epithelial–mesenchymal transition (Tian et al., 2017a; Zhang et al., 2019). These genes were enriched in H3K27Ac markers, characteristic of transcriptional elongation occurring via the superelongation complex (Tian et al., 2013). To address the question whether BRD4-dependent genes identified in this study were regulated by changes in chromatin accessibility, we analyzed the relationship between these data. In this analysis, we asked whether RSV-inducible ATAC-Seq peaks mapped to BRD4-dependent genes. 1,700 peaks were mapped to gene bodies (promoter, intron, exon, or UTRs) with the majority being mapped to promoter or intronic sequences (Figure 9A). Gene Ontology and pathway enrichment analyses indicated a substantial enrichment of genes controlling collagen fiber biosynthesis and/or formation (Figures 9B,C). These genes include ECM-regulating *MMP9*, cadherin-3 (*CDH3),* mucin 1 (*MUC1*), serpin family A member 1 (*SERPINA1*), and collagen type 17 alpha 1 chain (*Col17A1*).

**FIGURE 10:**
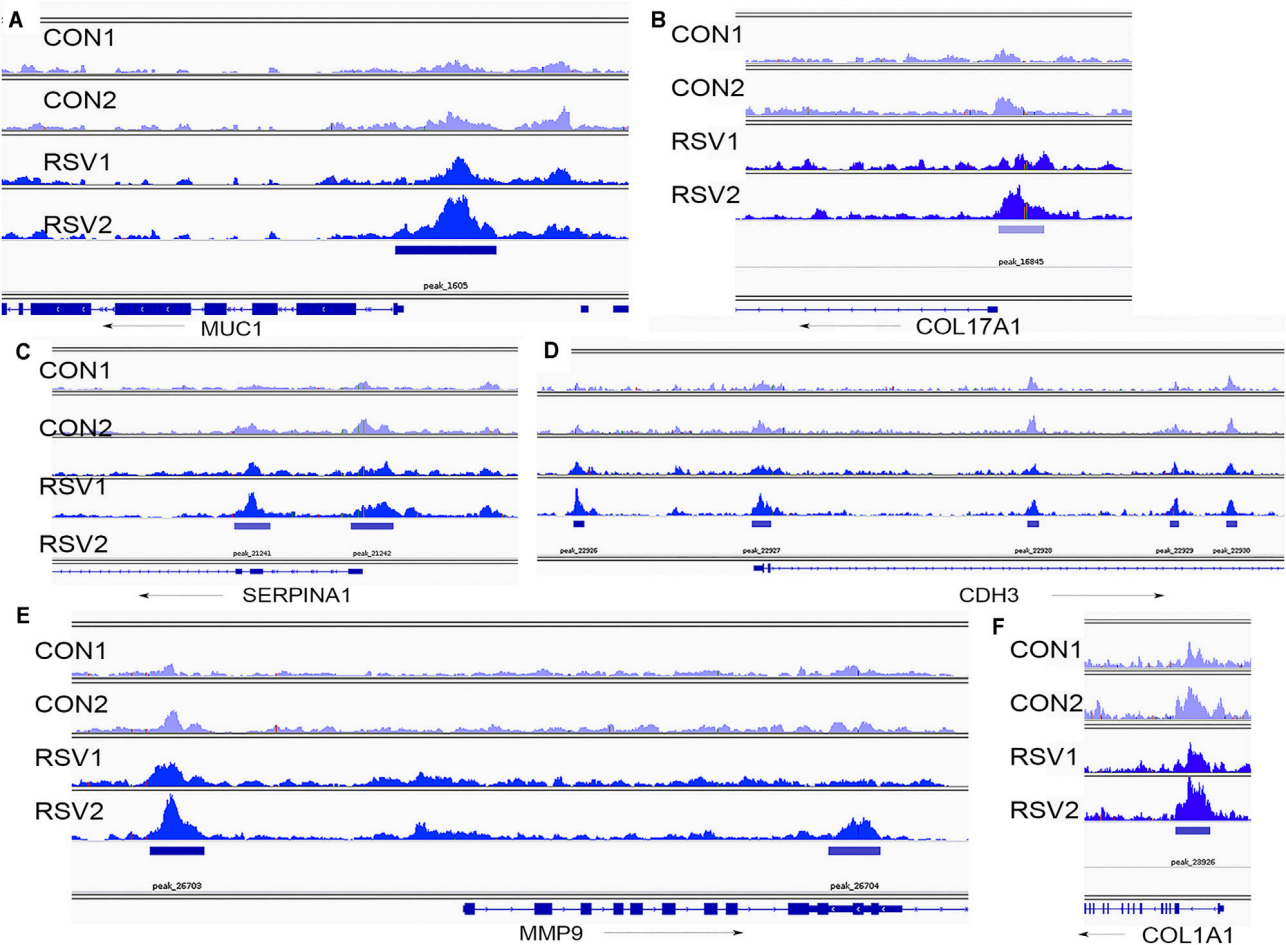
Chromatin-accessible peaks in 5′ regions of BRD4-dependent genes. Shown are individual ATAC-Seq tracks for replicate control and RSV-infected cells (Xu et al., 2020). **(A)**, *MUC1* locus; **(B)**, *COL17A1*, **(C)**, *SERPINA1*; **(D)**, *CDH3*; **(E)**, *MMP9*; and **(F)**, *COL1A1*. Direction of transcription is indicated by the arrow.

The effect of RSV and BRD4 inhibitors on MMP9 mRNA expression was independently validated in a separate experiment using qRT-PCR (Figure 9D). The mRNA expression profiles of C*DH3, MUC1, SERPINA1,* and *COL17A1* are shown (Figure 9E). ATAC-Seq peaks for these genes were visualized relative to the transcription start site using integrated genomics viewer, where a marked increase in genomic accessibility in 5’ regulatory regions is illustrated for each gene (Figure 10).

## Discussion

The airway mucosa is composed of discrete, highly differentiated airway epithelial cells that play an important role in gas exchange, maintenance of fluid balance, control of vascular reactivity, and initiation of the innate response (Crystal et al., 2008). Specialized cuboidal cells in the lower bronchiolar–alveolar junction are primed to rapidly respond to respiratory viral infections, through the elaboration of gene regulatory networks controlling mucosal inflammation and cell state changes (Harvey et al., 2007; Tian et al., 2018b). This coordinated genomic response is dependent on epigenetic processes, including chromatin remodeling and transcriptional elongation activity (Tian et al., 2016; Tian et al., 2017b; Tian et al., 2019b). Mechanistically, BRD4 associates with acetylated transcription factors activated by pattern recognition receptor binding, including NFκB/RelA and IRF to repositioning to networks of innate responsive genes. Despite a detailed understanding of the importance of BRD4 in promoter pause–release, the functional activities of BRD4 are incompletely understood. In this study, we apply highly selective BRD4 inhibitors to identify the role of BRD4 in the antiviral response to RSV. RSV replication in the lower airway epithelium triggers a genomic response important to the immunopathogenesis of this disease, including Th2 lymphocyte, myofibroblast expansion, and mucous obstruction (Zhao et al., 2017). Our robust RNA-Seq data extends the role of BRD4 as an activator and a repressor of the innate immune response, important in cytokine-induced airway remodeling and a subset of IFN genes. Our finding deduces that BRD4 is required for the autoregulation of its own mRNA as well as that of *RELA, MED* subunits, and *SMARC.* These findings extend the role of BRD4 to modifying enhanced coupling and nucleosomal phasing in this process.

### Bromodomain-Containing Protein 4 BD Is Required to Modulate IFN–IFN-Stimulated Gene Circuits

Because of the essential role of BRD4 in cell cycle transition, DNA damage repair and cell state transition, gene silencing approaches for BRD4 induce compensatory adaptations that result in the inference of the BRD4 difficult. Here, we employ an alternative approach using a highly potent and rapidly active competitive inhibitor of BRD4. Earlier, we reported on the discovery and validation of ZL0454 as a potent and selective inhibitor of BRD4 BD1 and BD2 domains, enabling the elucidation of the role of BRD4 in inducible signaling downstream of TLR3 (Tian et al., 2018a; Liu et al., 2018; Liu et al., 2020). ZL0454 binds in the acetylated lysine–binding pockets of BRD4 BD1 and BD2 with ∼80 nM affinity, forming hydrogen bonds with Asn140 directly and Tyr97 indirectly via a H_2_O molecule (Liu et al., 2018). Moreover, ZL0454 has 30-fold selectivity for BRD4 BDs over other BET family members, making this a unique reagent for probing the role of BRD4 in dynamic situations. That ZL0454 disrupts RSV-inducible protein interactions were shown in our recent affinity-mass spectrometry studies (Mann et al., 2021). In this study, we extend the functional understanding of these interactions on gene expression in the context of RSV infection.

Our previous study has shown that intracellular RSV replication is a profound activator of mucosal type I/III IFN expression (Jamaluddin et al., 2001; Liu et al., 2009). In the airways, virally infected cells sustain IFN production through a dramatic signal amplification process involving the IRF transcription factors and their upstream pattern recognition receptors, TLR3 and RIG-I (Tian et al., 2017b). IFNs play an important role in antiviral defense mechanisms by activating neighboring, uninfected cells, to express ∼300 downstream ISGs to promote an antiviral state and reduce viral spread (Smieja et al., 2008). Consequently, ISG expression is a dominant pathway seen in whole genome profiling of RSV-infected cells (Zhang et al., 2001; Zhang et al., 2003; Xu et al., 2020).

Our findings that ISGs *MX1, OASL,* and *IRF7* are inhibited by ZL0454 suggests that BRD4 is important in paracrine IFN signaling. We note that ISG inhibition cannot be attributed to the trivial effect that BRD4 inhibition artifactually reduces RSV replication, as enhanced expression of RSV patterns is seen in the ZL0454-treated cells (Figure 3A).Previous studies have shown no effect of BRD4 silencing or inhibition on RSV replication *in vitro* (Tian et al., 2017b). Moreover, the expression of the IFN antagonistic genes, NS1 and NS2, is similar in the ZL0454-treated cells.

The effect of BRD4 inhibitors on *MX1, OASL,* and *IRF7* is consistent with earlier mechanistic studies that BRD4 and the DRB sensitivity factor are rapidly recruited to ISGs after IFN stimulation (Patel et al., 2013) and RSV infection (Tian et al., 2018b). In both of these studies, BRD4 binding was enhanced by IFN/RSV, and this recruitment was reduced by the use of nonselective small molecule BRD4 inhibitors. These data suggest that the BD domain is involved in inducible promoter recruitment, either by disrupting binding to acetylated histones or acetylated transcription factors. Interestingly, not all IFNs are inhibited by ZL0454. For example, *IFNL3* is, in fact, upregulated by the administration of the inhibitor (Figure 4C), and *IFNβ* is not affected (not shown). These data indicate to us that IFN–ISGs are controlled by distinct transcriptional regulatory processes.

### Bromodomain-Containing Protein 4 Regulates Growth Factors and Extracellular Matrix Remodeling

*In vivo,* RSV infection triggers a process involving the expansion of subepithelial myofibroblasts important in extracellular matrix remodeling (Brasier, 2020). Our previous studies using ATAC-Seq identified that RSV produces nucleosome-free regions on *TGFB1/JUNB//FN1/MMP9* genes and the rate-limiting enzyme in the hexosamine biosynthetic pathway, glutamine-fructose-6-phosphate transaminase 2 (*GFPT2*), whose paracrine activity is critical in myofibroblast expansion (Xu et al., 2020). However, the mechanisms by how these chromatin remodeling events were produced were not elucidated. Our studies here showed that *IL-6* and *MMP9* expressions are highly BRD4-sensitive and indicate that BRD4 may play a critical role in chromatin opening in this growth factor pathway. Future studies identifying BRD4-sensitive RSV-induced chromatin domains will be illuminating.

### Bromodomain-Containing Protein 4 Autoregulation and Impact on the NFκB/RelA Pathway

Our studies are the first, to our knowledge, to indicate that BRD4 expression is inducible and autoregulated in response to RSV infection. The enhanced expression of *BRD4* mRNA, yet steady-state levels of BRD4 protein is reduced, indicates that BRD4 protein turnover increases on pathway activation. To this end, it is notable that the TRIM family of ubiquitin ligases have been identified in the BRD4 complex (Zhang et al., 2017). More research will be needed to examine the role of ubiquitination in BRD4-dependent gene expression.

On innate activation, NFκB/RelA undergoes a coupled phosphorylation/acetylation processing mediated by the IκB kinases and p300/CBP, respectively (Brasier et al., 2011; Nowak et al., 2008). BRD4 BD binds to the acetyl-K 310 residue of NFκB/RelA, (Figure 6G) and the complex is recruited to a subset of NF-κB and BRD4-dependent genes. Interestingly, PLA indicates that the presence of the RelA∙BRD4 complex is located in both cytoplasmic and nuclear fractions. These data are consistent with earlier immunohistochemical observations that BRD4 is primarily found in the nuclear compartment but also clearly detectable in the cytoplasm (see Figure 4 in (Tian et al., 2016)). This finding could suggest that the complex is initially formed in the cytoplasm, or the complex is actively shuttling in and out of the nucleus, consistent with real-time fluorescent imaging of activated RelA (Carlotti et al., 2000). Research by others has shown that BRD4 binding stabilized activated RelA in the nucleus. Upon disrupting BRD4 binding, nuclear acetylated RelA is rapidly degraded, and RelA is depleted in the cells (Zou et al., 2014). Our findings here suggest that the effect of BRD4 on NFκB abundance may also be mediated through the inhibition of NFκB gene expression. Collectively, these findings suggest that BRD4 inhibitors may profoundly modify inflammatory diseases.

### Bromodomain-Containing Protein 4 Modulates Mediators Subunit Expression

Mediators are a family of proteins that couple super enhancers to polymerases engaged on cell identity and oncogenes and whose actions are closely intertwined with those of BRD4 (Quevedo et al., 2019). Previous research in acute myelogenous leukemia cells have shown that BET proteins control the association of MED subunits with the MYB oncogene (Bhagwat et al., 2016). Interestingly, in this oncogenic context, the MED12, MED13, MED23, and MED24 subunits can functionally substitute for BRD4, producing BET inhibitor growth resistance. The MED complex is well established to interact with BRD4. In separate interactome studies, MED subunits are enriched with basal and activated BRD4 complexes (Zhang et al., 2017; Mann et al., 2021), and the interaction is disrupted by BD inhibition. Our findings that RSV regulates mRNA expression of individual subunits of MED complexes and that this regulation is modulated by BRD4 suggests that these modified complexes may have unique biological functions or target genes. More studies examining the role of changes in MED subunit expression on transcriptional elongation in the innate response will be of substantial interest.

### Bromodomain-Containing Protein 4–SMARC Interactions

This study indicates that BRD4 is required for sustained expression of *ARID1A* and *SMARC* subunits in RSV-infected airway epithelial cells. The SMARC proteins are a family of nonredundant ATP-dependent chromatin remodeling complexes that form cell-type specific complexes important in maintaining a differentiated epithelial cell phenotype (Xu et al., 2021b). Our study here indicates that the expression of many SMARC subunits is largely inhibited by RSV infection, SMARCB1, and its core ATP-dependent catalytic subunit SMARCA2/BRM prime IFNβ expression (Cui et al., 2004). We found earlier that SMARCA4 complexes are also important in priming IFNβ and IFNL mucosal interferons for a full antiviral response (Xu et al., 2021b). This mechanism involves enhanced accessibility of distal upstream enhancer/intergenic elements, perhaps suggesting MED interactions with proximal IFN promoters. The role of BRD4–SMARC expression may be important in maintaining 3D chromatin conformation and enhancer looping; these questions will require direct investigation.

### A Subset of Bromodomain-Containing Protein 4–dependent Genes Are RSV-Regulated by Chromatin Remodeling

Here, we provide evidence that a subset of the BRD4-dependent genes is associated with changes in chromatin accessibility. These genes are enriched in collagen RSV-induced formation and remodeling, consistent with our earlier findings that increased chromatin accessibility participates in RSV-induced cell-state transitions (Xu et al., 2020; Xu et al., 2021b). Although these inducible genomic sites are enriched in histone modifications associated with transcriptional elongation, it remains an open question whether these genes are regulated by transcriptional elongation or by enhancer-dependent transcription. Moreover, whether these sites are involved in BRD4 binding and/or regulated by BRD4 will require further investigation.

## Summary

**FIGURE 11:**
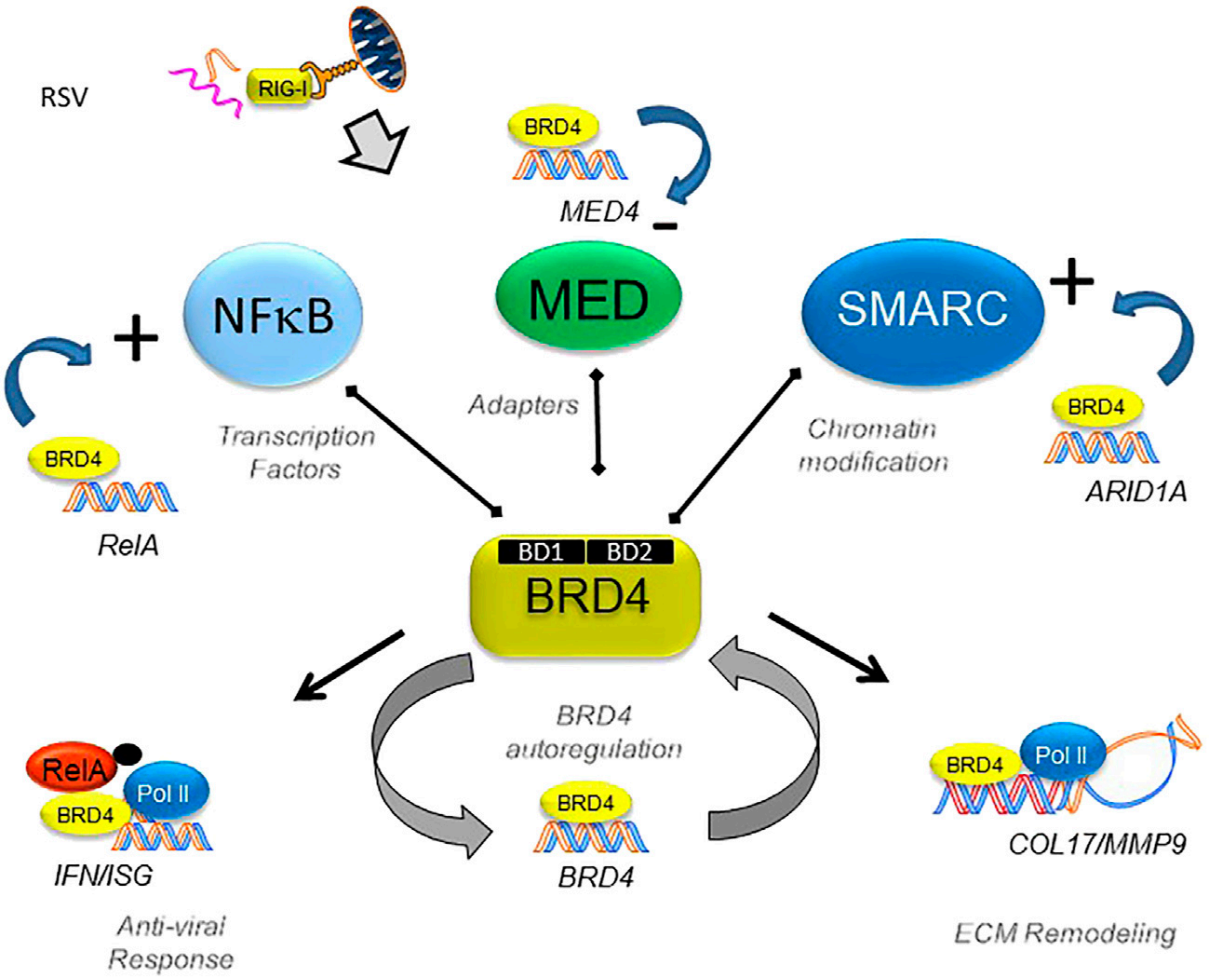
BRD4’s dynamically regulated gene networks. Schematic diagram of BRD4-dependent genes in RSV infection. Although BRD4 is required for innate signaling and expression of ECM remodeling proteins, this study provides evidence that BRD4 controls its own expression via an autoregulatory network. In addition, BRD4 controls the expression of members of its interacting coactivators that bind the BD, including transcription factors, adaptors, and chromatin remodeling complexes.

In the patterned epithelial innate immune response, BRD4 interacts with specific members of transcription factor families (NFκB), chromatin complex adapters (MED), and chromatin modifying factor (SMARC) to alter the activities of the complex which is schematically illustrated in Figure 11. Intriguingly, the expression of these interacting proteins is coordinately regulated by the antiviral host response. Using a selective, small-molecule BRD4 inhibitor, we demonstrate the dynamic role that BRD4 plays in the expression of its interacting coactivators. One of our surprising findings is that BRD4 participates in the RSV-inducible expression of RelA, in addition to its known effects on posttranslational stabilization. By contrast, BRD4 suppresses RSV-inducible expression of the MED subunits CDK12, CSRP1, ERCC3, MED17 and -27. By contrast, BRD4 activity is required for expression of the SMARC complexes, ARID-1A, -1B, SMARCAD1, and SMARCE1. In this way, BRD4 reshapes the protein-interacting complexes formed during the innate response to RSV. Moreover, our findings suggest that BRD4 is autoregulated and is actively consumed by the innate pathway. Integrating the results of the RNA-Seq and ATAC-Seq data, we identify the subset of BRD4-dependent genes that undergo RSV-inducible nucleosomal repositioning, resulting in enhanced transcription factor accessibility. This effect is in addition to the well-known effect of BRD4 complex interaction with innate response genes in chromatin-accessible domains (Brasier, 2020; Brasier et al., 2011; Tian et al., 2013). We note that genes undergoing chromatin transition into an open configuration are enriched for those involved in ECM formation and remodeling (Figure 11). Our study, therefore, extends the understanding of how BRD4 inhibitors function as effective anti-viral therapeutics by affecting virus-inducible ECM remodeling (Brasier, 2020).

### Therapeutic Implications

Repetitive viral infections producing acute exacerbations of asthma or chronic obstructive pulmonary disease (Tuffaha et al., 2000; Gern, 2015) are linked to reduction in pulmonary function (Calhoun et al., 2015). Using a model of repetitive viral inflammation mediated by TLR3, we demonstrated that BRD4 is a central mediator of airway remodeling, coupling the innate inflammatory pathway to epithelial cell-state changes and fibrosis by the expansion of subepithelial fibroblasts (Tian et al., 2018a). Consequently, BRD4 is a validated target for the treatment of airway remodeling in viral and allergic lung diseases (Brasier and Zhou, 2020). Through its association with inflammation-activated NFκB/RelA, BRD4 is dynamically repositioned to growth factor and ECM genes, resulting in innate training, epithelial cell-state transition, and secretory myofibroblast transdifferentiation (Ijaz et al., 2017; Yang et al., 2017; Brasier, 2019). In particular, the epithelial *TGFβ/IL6/MMP9* pathway plays an important role in triggering myofibroblast expansion (Xu et al., 2020; Xu et al., 2021b). These genes undergo changes in chromatin accessibility in response to RSV infection by largely unknown mechanisms. This study extends the mechanisms of how BRD4 inhibitors disrupt inducible cytokine–growth factor circuits by disrupting BRD4 binding to chromatin and by inhibiting the expression of coactivators important in inflammation and remodeling.

## Data Availability

The datasets presented in this study can be found in online repositories. The names of the repository/repositories and accession number(s) can be found below: https://www.ncbi.nlm.nih.gov/geo GSE179353.
